# Pharmacological and physiological effects of cannabidiol: a dose escalation, placebo washout study protocol

**DOI:** 10.1186/s12883-024-03847-1

**Published:** 2024-09-12

**Authors:** J. Patrick Neary, Jyotpal Singh, Jane Alcorn, Robert B. Laprairie, Payam Dehghani, Cameron S. Mang, Bruce H. Bjornson, Thomas Hadjistavropoulos, Holly A. Bardutz, Lanishen Bhagaloo, Zachary Walsh, Michael Szafron, Kim D. Dorsch, Elizabeth S. Thompson

**Affiliations:** 1 Faculty of Kinesiology & Health Studies, University of Regina, 3737 Wascana Pkwy, Regina, SK S4S 0A2 Canada; 2 Cannabinoid Research Initiative of Saskatchewan (CRIS), Saskatoon, SK Canada; 3 Department of Cardiology, Prairie Vascular Research Inc, Regina, Canada; 4 College of Pharmacy and Nutrition, University of Saskatchewan, Saskatoon, Canada; 5 College of Medicine, University of Saskatchewan, Saskatoon, Canada; 6 University of British Columbia, Vancouver, Canada; 7 Department of Psychology, Centre of Aging and Health, University of Regina, Regina, Canada; 8 Department of Psychology, University of British Columbia, Kelowna, Canada; 9 School of Public Health - Biostatistics, University of Saskatchewan, Saskatoon, Canada

**Keywords:** Cannabidiol, Neurophysiology, Pharmacokinetics, Cardiovascular physiology

## Abstract

**Background:**

Cannabinoids such as cannabidiol (CBD) exhibit anti-inflammatory properties and have the potential to act as a therapeutic following mild traumatic brain injury. There is limited evidence available on the pharmacological, physiological and psychological effects of escalating CBD dosages in a healthy, male, university athlete population. Furthermore, no dosing regimen for CBD is available with implications of improving physiological function. This study will develop an optimal CBD dose based on the pharmacokinetic data in contact-sport athletes. The physiological and psychological data will be correlated to the pharmacokinetic data to understand the mechanism(s) associated with an escalating CBD dose.

**Methods/design:**

Forty participants will receive escalating doses of CBD ranging from 5 mg CBD/kg/day to 30 mg CBD/kg/day. The CBD dose is escalated every two weeks in increments of 5 mg CBD/kg/day. Participants will provide blood for pharmacological assessments at each of the 10 visits. Participants will complete a physiological assessment at each of the visits, including assessments of cerebral hemodynamics, blood pressure, electrocardiogram, seismocardiogram, transcranial magnetic stimulation, and salivary analysis for genomic sequencing. Finally, participants will complete a psychological assessment consisting of sleep, anxiety, and pain-related questionnaires.

**Discussion:**

This study will develop of an optimal CBD dose based on pharmacological, physiological, and psychological properties for future use during contact sport seasons to understand if CBD can help to reduce the frequency of mild traumatic injuries and enhance recovery.

**Trial registration:**

Clinicaltrials.gov: NCT06204003.

## Background

Plant-based (phyto) cannabinoids such as cannabidiol (CBD) and Δ^9^-tetrahydrocannabinol (Δ^9^-THC) have been generating increasing research interest due to their proposed therapeutic properties. These include pain relief, neurogenesis and reduced inflammation [1, 2]. The short- and long-term effects of brain injury, especially sport-related concussion (SRC), or mild traumatic brain injury (mTBI), pose serious challenges for our health care system. Currently, there are gaps in the literature related to the medical benefits of using CBD for this clinical population. Key gaps include information regarding safety of daily consumption, optimal dosage for physiological function, possible neuroprotective effects, and whether they can be used to reduce prescription medications, including opioids, for chronic pain management [3].

While there is a theoretical framework for CBD use for brain injury, the evidence is currently limited [4–8]. Sport-related concussions appear to induce stress on the entire body, with alterations in heart function, immune responses, and cerebral hemodynamic activity [9–12]. Specifically, impairments in heart rate variability, baroreflex sensitivity, blood pressure variability, cerebral blood flow velocity, and cerebral oxygenation can occur during acute concussion and in those with a history of concussions [13–19]. Therapeutic options are currently limited, and recently, there has been interest in treating brain injuries with cannabinoids such as CBD, with an emphasis on concussion [4]. Recognition that the endocannabinoid system (eCB) system regulates neurotransmission and inflammatory processes in part through the type 1 and 2 cannabinoid receptors (CB1 and CB2) [8, 20], prompted investigations into how phytocannabinoids could be used to modulate neurotransmitter release and inflammation [21], ultimately leading to neuroprotection [22, 23]. As such, understanding how different CBD doses influence physiological and pharmacological parameters will allow us to provide an optimal dose for future research to administer in a competitive sports season for enhanced neuroprotection.

CBD appears to work via a number of receptors, including non-cannabinoid receptors, such as the G-protein-coupled receptors serotonin 1A and adenosine A2a, the ligand-gated vanilloid receptor ion channels, and the nuclear peroxisome proliferator-activated receptors [4, 24]. Collectively, modulation of these receptors can result in extensive anti-oxidative and anti-inflammatory effects, along with influencing hemodynamic activity [4, 25, 26]. Consequently, CBD has the potential to limit blood brain barrier permeability, improve cerebral functional connectivity, and the heart-brain axis interactions following mTBI [4, 24]. To date, no research has examined CBD-mediated neuroprotection in elite contact sport athletes, but such effects have been inferred [3, 4, 27]. Furthermore, specific dosage, pharmacokinetic, and pharmacodynamic data are lacking, or are difficult to compare between studies that use different sources of CBD [28].

This protocol presents the first of a three-part research project aimed at investigating the pharmacological, physiological, and psychological effects of CBD in healthy, young (18–35 years) males. Female participants are excluded as the study needs to be directly applicable to the National Football League (NFL) players due to the funding requirements, requiring our sample to be male. We also predominantly recruited participants that played American style Football. However, our future research will include a similar study in female participants participating in contact sports. The primary objectives and endpoints are to develop an optimal CBD dose based on the pharmacokinetic data in contact-sport athletes. The secondary objectives include direct neurophysiological and pharmacological analyses to understand the mechanism(s) associated with the primary objective findings. The primary research hypothesis is that CBD formulations are non-intoxicating (non-psychotropic), safe, well-tolerated and do not cause adverse physiological or psychological dysfunction. Specifically, a placebo-washout, dose escalation Phase I clinical trial will be conducted to document the effects of increasing dosages of CBD on pharmacokinetic and pharmacodynamic parameters to determine what is an ‘optimal’ formulation for daily administration for physiological function. This dose will be applied to future research studies to understand the influence of CBD on neuroprotection from concussion. The secondary hypotheses are:

1. Plasma levels of CBD and/or its active metabolites will correlate with cerebrovascular, neurophysiology and cardiovascular physiology outcome variables.
2. Saliva levels of CBD and/or its active metabolites will correlate with plasma samples.

## Methods and design

### Study design

**Fig. 1:**
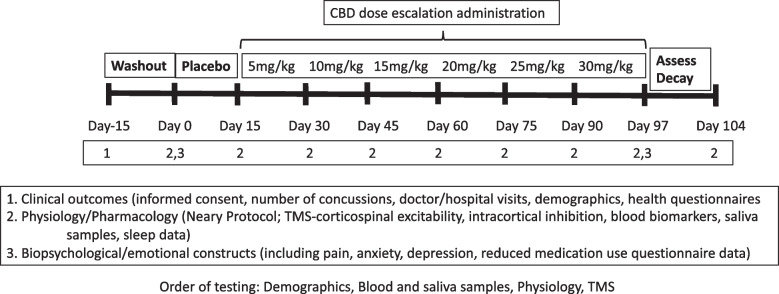
Overview and Schedule for the Study Protocol. Dosing regimen and assessments to be completed at each visit. Neary protocol derived from [36]

We will complete a two-centre, phase 1, open-label dose escalation study to determine the safety and tolerability of CBD in healthy male athletes. The study will be conducted in two centres in Canada (Regina and Saskatoon, SK). The study consists of a baseline phase, a treatment phase, and a post-dosing phase. Figure 1 illustrates the study visits.

### Study population

We will recruit 40 participants across two study sites. Eligibility to participate in the study include the following criteria:

1. Healthy male adults between 18–35 years of age that compete (in any level, including recreational, collegiate, professional) in contact sport athletics (including American football, soccer, rugby, wrestling, and hockey).
2. Non-smokers without known medical complications.
3. Ability to maintain commitment to all proposed biopsychological and health questionnaires, and neuro-physiological and physiological laboratory tests.

Participants meeting any of the following criteria are excluded from the study:

1. Currently under treatment (e.g., by a physician or clinical psychologist) for anxiety, depression, post-traumatic stress disorder, mood disorder, psychotic disorders or any cerebrovascular and cardiovascular complications.
2. Unable to maintain commitment to all proposed biopsychological and health questionnaires, and neuro-physiological and physiological laboratory tests.

Since the study is employing a continuous dose escalation design, a placebo-washout design will be implemented. All participants will first intake a placebo for 2 weeks prior to beginning their CBD dosing protocol. As such, no participant randomization of participants will occur, and neither the study participants nor the study investigators will be blinded to the CBD dose. As we will likely recruit the majority of the participants from the university population, they are expected to be in their off-season.

### Intervention

Study participants will receive escalating doses of CBD ranging from 5 mg CBD/kg/day to 30 mg CBD/kg/day. The CBD dose is escalated every two weeks in increments of 5 mg CBD/kg/day. The investigational product is a 99% CBD isolate (GVB Biopharma, OR, USA) using conventional distillation and extraction industry techniques under Good Manufacturing Practices (GMP). The product is regulated by the Access to Cannabis for Medical Purposes Regulations under the supervision of Health Canada's Good Manufacturing Practices. Health Canada requires any cannabis product to be free from molds, mycotoxins, and pesticides and that the concentration of CBD is verified by an independent third-party laboratory.

### Procedures

#### Baseline Phase

##### Visit 1 (Day -15): Consent

Participants who meet the eligibility criteria and have signed the informed consent will be enrolled into the study. From each participant, a medical history (including demographics such as height, body mass), health questionnaires (paper and pen), and a saliva sample will be taken. Participants will receive a logbook to record any side effects associated with the study product during the treatment phase. This session will take approximately 20–30 min to complete. The body mass recorded at this initial visit will be used for the dosing regimen.

Participants will be asked to refrain from taking Cannabis or Hemp products between study Visit 1 and 2 (approximately 2 weeks later) to ensure undetectable cannabinoid levels in their blood at the commencement of the treatment phase. As well, participants will be instructed to not smoke, use chewing tobacco, or use any Cannabis or Hemp products besides the study CBD for the duration of study. These are important requirements to continue in this study. Participants will be asked to not complete Visit 1 and to terminate the study if they feel that they cannot adhere to these requirements.

##### Visit 2 (Day 0): Baseline Tests

Participants will provide blood (to confirm that participants do not have measurable cannabis products in their bloodstream) and saliva samples, complete the biopsychological and health questionnaires, and perform the neuro-physiological and physiological laboratory tests. This entire session will take approximately 2–3 h.

At the end of Visit 2, participants will be given a 16-day supply of study Placebo to be taken orally over the next 2-weeks. The Placebo (cornstarch maltodextrin powder) will be supplied as appropriately labelled, individual dosing units (glass vials), and participants will be provided with a hand-out that explains the administration of the placebo. A small amount of extra Placebo will be provided to allow for some flexibility in scheduling the next appointment. Participants will be instructed to take an individual dosing unit twice per day, once in the morning at breakfast and once at prior to sleeping at night, with an aim for each dose to be 12 h apart. The study placebo will be taken with 250 mL of water and a Blender Bites smoothie ‘puck’ and given to participants by one of the researchers conducting the study. Further, the participants will be asked to record any adverse effects into the Logbook provided to them on Visit 1.

##### Treatment Phase

Visit 3–9 (Days 15—97).

At Visit 3 (Day 15) participants undergo the same laboratory testing procedures as conducted during the baseline assessments. They will be told to discontinue the Placebo product and will receive the CBD from a study researcher. The CBD product will be supplied as appropriately labelled, individual dosing units (glass vials) and participants will be provided with a hand-out that explains the administration of the CBD product. The first CBD dose at that particular dosing level will be administered to the participant. Participants will be instructed to take an individual dosing unit twice per day, once in the morning at breakfast and once in the evening approximately 10 – 12 h since the morning dose. The study drug will be taken with 250 mL of water and a Blender Bites smoothie ‘puck’ (https://blenderbites.com/). Further, the participants will be asked to record any adverse effects into the Logbook provided to them on Visit 1.

After 2 weeks of treatment, participants will return to the study laboratory for follow-up testing that includes blood and saliva sampling, biopsychological and health questionnaires, and neuro-physiological and physiological laboratory tests. They will return all empty and unused individual dosing units to allow the study research personnel to determine compliance. They will be told to discontinue the current CBD dosing level and will receive a supply of higher dose CBD product according to the dose escalation schedule. Blood samples will be taken prior to the next CBD dose level administration to allow determination of plasma concentrations of CBD and its metabolites prior to increasing the dose. Immediately following the blood sampling, the participant will be administered the new CBD dose level. Participants will be asked to update the researchers regarding any symptoms or adverse events, and to record these in their Logbook. Visit 9 represents the end of the treatment phase. Participants will discontinue the CBD product and will return all empty and unused samples to the study researchers.

##### Post-dosing Phase

Visit 10 (Day 104; End of study).

Participants will return to the research laboratory for final testing, which will include collection of blood and saliva samples, completion of biopsychological and health questionnaires, and neuro-physiological and physiological laboratory tests. This testing session will help us to re-assess participants to determine the manner in which the study product remains in their body, as shown by their blood results, after terminating its use. Logbooks will be collected at this time.

### Withdrawal criteria

Study participants may withdraw from the study at any point. Participants will be withdrawn if they suffer from any intolerable adverse effects resulting from the CBD intervention, if they fail to attend required site visits, or they are noncompliant with the CBD dosing regimen. For transparency, though, all participants withdrawn from the study will be included in the final report.

### Sample size justification and statistics

Due to the limited research available, we will assume a 0.5 correlation between 10 successive data time points. To detect a 0.25 moderate effect size in cardiovascular metrics (blood pressure and heart rate variability) with 90% power and 95% confidence, 36 total participants are required. To account for dropout and non-compliance rates, 15–20% more participants will be recruited in each study. Statistical analysis will be performed using IBM® SPSS® Advanced Statistics, and a combination of statistical analyses will be used depending on the outcome variable. In general, multiple repeated multilevel linear mixed models will be used with statistical significance set at p ≤ 0.05.

### Physiological and psychological testing

Venous blood samples will be taken at each visit to monitor the safety of the cannabinoid intervention in each study and/or to conduct pharmacological assessments. A licensed medical laboratory technician will complete a blood draw via venipuncture from the cubital fossa. The blood samples will be analysed by an accredited provincial laboratory for basic hematological and clinical chemistry parameters, and by the Dr. Jane Alcorn’s laboratory in the College of Pharmacy & Nutrition at the University of Saskatchewan for quantitative determination of cannabinoid plasma levels. This laboratory will also conduct the analysis of biomarkers using commercially available kits.

1. Blood collected in a lithium heparin tube will allow for assessment of CBD and Δ^9^-THC concentrations and their active metabolites. These will allow us to study the pharmacokinetics and determine the relationship among plasma levels, efficacy and side effects.
2. Blood collected in a serum separating tube will allow for assessment of biomarkers that include Tumor Necrosis Factor alpha (TNF-alpha), brain-derived neurotrophic factor (BDNF), Interleukin-6, and C-reactive protein. These markers will complement our physiological parameters and will be analyzed using immunoassay ProcartaPlex™ kits performed on a Luminex™ platform (Life Technologies, Frederick, MD) as per other studies [29].
3. Blood collected in a lithium heparin tube will allow for genome-wide sequencing analysis for single nucleotide polymorphisms and copy number variants (*e.g.* CNVs for cannabinoid-metabolizing enzymes such as cytochrome P450 2D6 *CYP2D6*) (Next Generation Sequencing Laboratory, University of Saskatchewan, Saskatoon, Saskatchewan, Canada).
4. For initial risk assessment and safety, blood collected in a serum-separating tube and EDTA tube will be analyzed at the Regina General Hospital laboratory for assessment of complete blood count (CBC) and differential, and assessment of general chemistry (such as liver enzymes).

All blood tubes are 4–6 mL each. A small amount of the blood will be kept frozen for future research related to this study if participants indicate agreement on the consent form. In addition to the blood samples, saliva samples will be collected. This will allow for DNA analysis for metabolic genes [30], and will be analyzed by My Next Health Inc. (Oakville, Ontario, Canada). The salivary are processed through an automated DNA Extraction Pipeline. After extraction is completed, each sample undergoes’ genomic DNA quality control, and the passing samples proceed to sample preparation. Sample preparation converts a sample into a library of fragments which is sequenced on a Next-generation sequencing (NGS) instrument. The Nucleotides are “read" on the Illumina NovaSeq 6000 sequencer at an 80X depth/read number to ensure appropriate accuracy. Finally, the genes and gene variants are called using the Illumina DRAGEN pipeline and confirmed by the My Next Health (https://mynexthealth.com) data-driven QAQI analysis.

### Sleep quality assessment

As sleep quality and sleep efficiency (time actually asleep compared to time in bed) are also altered following a sport-related concussion [31, 32], and in those with persisting post-concussion symptoms/symptomology (PPCS) [33], all participants will be given an actigraph (Phillips Respironics, Actiwatch) to wear to bed each night. The actigraph records details about sleep, such as time spent in bed, time spent asleep, and wake after sleep onset. Furthermore, participants will complete the Leeds Sleep Evaluation Questionnaire at each laboratory visit [34]. Research pertaining to the influence of CBD and ∆^9^-THC on sleep is still in its infancy, however, there is still some evidence available to show improvements in sleep quality following administration of these cannabinoids [35].

### Protocol

#### *(1) Cerebrovascular and cardiovascular protocol*

Each study will require participants to complete this “Neary” protocol [36] to understand how their cerebrovascular and cardiovascular function are affected by intake of the cannabinoids. Prior to testing, participants will be asked to refrain from consuming any alcohol for 24 h, from exercising for 12 h before, and from consuming caffeine for 6 h [37]. The participants will be encouraged to eat a small meal at least 2 h before testing and asked to void their bladder within 30 min of arrival. Participants will be attached to the following equipment: bilateral transcranial Doppler to measure blood flow velocity of the brain [19, 38], Functional near infrared spectroscopy (fNIRS) – fNIRS to measure oxygenation of the brain [13], and a Finapres Nova to record electrocardiogram and blood pressure continuously via finger photoplethysmography. The Finapres has a 5-lead ECG to record heart rate and the electrocardiogram [36, 39], a mouth-piece connected to a gas analyzed to collect expired carbon dioxide and oxygen [40], and a seismocardiograph to record cardiac cycle timing intervals and contractile parameters [12, 41]. After participants are attached to the equipment, they will complete the following protocol:

1) 30-min supine rest to establish spontaneous physiological baseline [42]

This protocol can provide insights associated with different peak activity intensities within six frequency intervals, each of which is associated with the following physiological functions: cardiac interval (0.6–2 Hz), respiratory interval (0.145–0.6 Hz), smooth muscle cell activity interval (0.052–0.145 Hz), autonomic innervation of smooth muscle (0.021–0.052 Hz), endothelial function related (0.0095–0.021 Hz), and nitric oxide dependent (0.005-0.0095 Hz) intervals. The activity intensities of cerebral blood flow velocity, blood pressure, and fNIRS parameters will be calculated at each interval during the 30-minute supine rest. This allows for analysis of resting state networks, such as the default mode network. During the first minute, the seismocardiograph recording (i.e., seismocardiogram) will be completed.

2) Six-breaths/minute paced breathing maneuver repeated for 5-min [18]

A 6-breaths per minute pace has been shown to allow for comparable measurements of autonomic function, including heart rate variability, baroreflex sensitivity, and blood pressure variability, and allows for a similar adaptation of respiratory sinus arrhythmia during each assessment.

3) Two-minute washout period to re-establish baseline physiology.

4) Squat-stand baroreflex maneuver (10 s squat:10 s stand, repeated 5-min) to assess flow-pressure reactivity and dynamic cerebral autoregulation.

Repeated squat stands is a useful method of assessment of dynamic cerebral autoregulation. This method have been used to study parasympathetic baroreflex responses, blood pressure alleviation, cerebral oxygenation, and cerebral blood flow responses [15, 43–45].

This complete protocol will take ~ 45 min.

#### *(2) Neurophysiological assessment protocol*

Transcranial magnetic stimulation (TMS) – TMS will be used to assess δ-aminobutyric acid (GABAergic) inhibition in the primary motor cortex [46, 47]. TMS will be delivered with a figure-eight coil and guided by neuro-navigation software to target the cortical motor representation for the first dorsal interosseous muscle of the dominant hand, from which surface electromyography (EMG) will be recorded. Standard procedures will be followed to identify the corresponding hotspot, and resting and active motor thresholds (rMT, aMT). To evaluate cortical inhibition, two TMS measures will be obtained: the cortical silent period (CSP) and short-interval intracortical inhibition (SICI). To elicit the CSPs, single-pulse TMS will be delivered at 120% of aMT while participants maintain a pinch grip contraction generating 20% of maximum voluntary contraction force. The duration (ms) of the transient quiescence in EMG activity following the motor evoked potential will be measured as the CSP. To assess SICI, we will deliver paired-pulse TMS. In this protocol, a sub-threshold conditioning stimulus will precede a supra-threshold ~ 1 mV amplitude test stimulus by 2 ms. The reduction in amplitude of the test stimulus when ‘conditioned’ by the preceding stimulus will be measured as SICI. To ensure valid comparison of paired-pulse measures across different time-points, where necessary we will adjust the test stimulus, so that test MEP sizes will be matched to within 30% of the comparison time-point. For each of the aforementioned measurements, 20 pulses of TMS will be delivered with inter-stimulus intervals of 5–8 s.

This protocol will take approximately 30 min to complete.

#### (3) Health questionnaires and nonverbal pain behavior

A key component of our analysis is the impact of our CBD administered continuously on normal psychological function. Thus, a number of health questionnaires will be administered to assess mental health over the duration of the study. These will include an initial medical history (including medication information) form, the Health-related quality of life (HRQOL), the Generalized Anxiety Disorder (GAD-7) Questionnaire, the Brief Pain Inventory-Short Form (BPI-SF), McGill Pain Questionnaire-Short Form (MPQ-SF), Numeric Rating Scale for Pain (NRS), and Genotype/phenotype Questionnaire. Complementing this self-report, we will use a validated Pain Behaviour Measurement system (PBM) [48] to capture non-verbal pain expressions during movement, where applicable [49]. Additionally, we will complete the sport concussion assessment tool 6 (SCAT6) symptom checklist at each visit, which includes extensive details pertaining to previous history of concussion on the initial intake.

Our assessment approach is consistent with the Initiative on Methods, Measurement, and Pain Assessment in Clinical Trials recommendations [50, 51] and involves assessment tools that are widely validated across pain types and populations.

#### (4) Pharmacokinetic assessment

This assessment aims to determine whether a correlation exists between dose, steady-state trough concentrations (C_SS,Min_), and/or pharmacodynamic assessment for the cannabinoids and their active metabolites. To draw such correlations, blood samples must be collected just before a dose administration when the concentrations are at steady state. Plasma levels of CBD and its metabolites will be assessed using a previously described validated Liquid Chromatography-Mass Spectrometry (LC–MS/MS) method with minor modifications [52]. Heparinized lithium Barricor vacutainers ® (BD Canada, Mississauga, ON) will be used to collect blood samples from the study participants [53]. Following collection, venous blood will be centrifuged at 1500 rpm for 10 min, and plasma transferred to LoBind microcentrifuge tubes and stored at − 80 °C until analysis.

The validated LC–MS/MS method can quantify plasma concentrations of THC and CBD and their active metabolites, 11-OH-THC and 7-OH-CBD, respectively. Stock solutions (in methanol) of 1 mg/mL of cannabinoids and respective stable isotope-labeled internal standards (Cerilliant Corp., Round Rock, TX) will be stored at -20 °C until use. To create a calibration curve, the stock solutions will undergo serial dilutions using blank human plasma. Low, medium, and high concentration Quality Control standards (two at each level) will determine the acceptance criteria for the analytical run, and will be incorporated into each analytical run of participant samples. A plot of the peak area ratios of the cannabinoid and its respective internal standard against the nominal concentration will construct the calibration curve. A linear least-squares regression analysis using 1/X as a weighting factor will determine the linearity of the calibration curves with requirement that all calibration curves have a coefficient of determination (r^2^) > 0.99. Intra- and inter-day precision and accuracy will also be within 15%, except at the limit of quantification that will be within 20%, and all Quality Control samples for a given run will be within 15% of their nominal values. Preparation of participant plasma samples will involve addition 200 μL cold acetonitrile and 10 μL internal standard working solution, followed by vortex-mixing and centrifugation for 10 min at 20,000 g while maintaining a temperature of 4 °C. Supernatant will be transferred to borosilicate tubes and dried under filtered air for 30 min at 37 °C. Samples will be reconstituted with 200 μL mobile phase and 100 μL transferred into HPLC inserts.

Quantification of the cannabinoids will be performed using an Agilent 1290 binary pump LC system with an online degasser (Mississauga, ON) coupled to an ABSciex 6500 triple quadrupole mass spectrometer with TurboSpray ionization (Concord, ON). 5 μL will be injected onto a Zorbax Eclipse XDB-C8 narrow bore (2.1 × 12.5 mm, 5 μm) guard column and Zorbax Eclipse XDB-C18 small-bore (2.1 × 12.5 mm, 5 μm) column while maintaining the column temperature at 30 °C to separate the cannabinoids. Mobile phase A (water containing 0.1 mM ammonium formate) and B (methanol containing 0.1 mM ammonium formate) at a flow rate of 250 μL/min and a 10 min gradient will separate the cannabinoids. Positive ion mode electrospray ionization (ESI), with an ion spray voltage of 5500 V, will be used to conduct multiple reaction monitoring (MRM) for quantification of CBD (quantifier ion m/z 315.1 > 193.2; qualifier ion m/z 315.1 > 259.2), THC (quantifier ion m/z 315.1 > 193.1; qualifier ion m/z 315.1 > 259.2), 11-OH-THC (quantifier ion m/z 331.1 > 193.1; qualifier ion m/z 331.1 > 201.0), CBC (quantifier ion m/z 315.1 > 193.2; qualifier ion m/z 315.1 > 259.2), CBD-d3 (318.2 > 196.1), THC-d3 (318.2 > 196.1), and 11-OH-THC-d3 (334.2 > 196.1) using a ABSciex 6500 QTRAP mass spectrometer (ABSciex, Concord, ON, Canada) and MultiQuant 3.0.1 Software. The temperature, gas source 1, gas source 2, curtain gas, entrance potential, and collision activated dissociation gas were set to 600 °C, 70, 60, 50, 10 V, and 10, respectively. The samples will be analysed in batches within three months of collection as excellent stability has been confirmed only for up to 3 months when stored at -80 °C [52].

### Medications/treatments permitted during the trial

Psychotropic medications with serotonergic activity will not be permitted during the study. Smoked cannabis can also enhance the effects of narcotic medications such as codeine, morphine and oxycontin. However, we will use a high CBD formulation that is suggested to be minimal risk and is not expected to adversely interact with these medications [54]. Therefore, if participants are taking these medications on a regular basis they will be allowed enrolment in this study but will be monitored by the Qualified Investigator and Principle Investigator of these studies.

### Monitoring of subject compliance

The graduate student researchers who will make weekly phone calls and monitor and encourage logbook completion will monitor participant compliance. Participants will be asked to return all unused study medication in the provided container at each visit as a measure of drug accountability and participant compliance. All unused study medication will be recorded.

### Safety parameters

Participant safety will be assessed using the following parameters:

- Quality of life questionnaire at Visits 1 and appropriate follow-up visits (Fig. 1).
- Laboratory testing conducted at Visit 2 and follow-up visits (Fig. 1)
- Adverse Events monitored and recorded at Visits 2 all subsequent visits. The rating scale includes items related to sleepiness/lethargy, irritability, nausea/vomiting and diarrhea.

### Adverse events

According to the International Council for Harmonisation of Technical Requirements for Pharmaceuticals for Human Use (ICH) guidelines for Good Clinical Practice, an adverse event is any untoward medical occurrence in a participant or clinical investigation participant when administered a pharmaceutical product that does not necessarily have a causal relationship with this treatment. An adverse event can therefore be any unfavorable and unintended sign (including an abnormal laboratory finding), symptom, or illness temporally associated with the use of a medicinal (investigational) product, whether or not related to the medicinal (investigational) product.

### Serious adverse events (Immediately Reportable to the Sponsor)

A serious adverse event is any adverse event that meets any of the following criteria:

- Fatal (i.e., the adverse event actually causes or leads to death);
- Life threatening (i.e., the adverse event, in the view of the investigator, places the participant at immediate risk of death). This does not include any adverse event that had it occurred in a more severe form, or was allowed to continue, might have caused death;
- Requires or prolongs inpatient hospitalization;
- Results in persistent or significant disability/incapacity (i.e., the adverse event results in substantial disruption of the participant’s ability to conduct normal life functions);
- Congenital anomaly/birth defect in a neonate/infant born to fathers exposed to study medication;
- Significant medical event in the investigator's judgment (e.g., may jeopardize the participant or may require medical/surgical intervention to prevent one of the outcomes listed above).

Study Investigators will seek information on adverse events at each participant contact. All adverse events, whether reported by the participant or noted by study personnel, will be recorded. During and following a person's participation in a trial, the investigator/institution should ensure that adequate medical care is provided to them for any adverse events, including clinically significant laboratory values, related to the trial. The investigator/institution should inform a participant when medical care is needed for current illness(es) of which the investigator becomes aware. The study investigators will be responsible for ensuring that all adverse events are recorded and reported to the Sponsor in accordance with the adverse event reporting instructions. Specifically, if the PI and QI agree that the staff-identified adverse event is serious, this will be reported to Health Canada by the Sponsor as per Health Canada regulations, and to the Research Ethics Boards by Site Investigators. Should the serious adverse event be unexpected (SUSAR) and related to the CBD, a report will be sent to Health Canada and the Research Ethics Boards within 15 days. Based on this report, if amendments are required to the informed consent form, this process will be completed and all current participants will be notified of this potential adverse event and be required to re-consent and complete the informed consent process again.

### Direct access to source data/documents

The Investigator acknowledges that some authorities have a duty to monitor the study records to make sure all the information is correct. The study records may be inspected under the authority of the investigator or his qualified designate by representatives of the Sponsor, Health Canada or the Research Ethics Board, as necessary.

### Ethics and dissemination

All information collected on the data collection form will be de-identified and no direct personal identifiers will be recorded. All measures will be taken to maintain confidentiality on collected data. A list connecting the participant’s name to the participant serial number listed on the data collection form will be stored on a password-protected computer and access to this list will be limited to research staff directly involved in the project. As per the Health Canada requirements, data will be stored for 15 years, and the Sponsor shall maintain all records related to the study for a period of 15 years. All data will be de-identified and only aggregated data will be presented at conferences and published in peer-reviewed journals. The data will be stored locally with the investigators.

### Compensation

Participants will each be compensated $250 for completing the entire study. If participants elect to withdraw from the study, they will be paid in proportion to the amount of time that volunteered to participate.

## Human ethics and consent to participate declarations

Ethics approval has been attained for this study from the University of Saskatchewan Research Ethics Board (REB Bio#3455). Health Canada approval (No Objection Letter) was obtained for this clinical trial study, and this study is registered with ClinicalTrials.gov (ID NCT06204003). All participants enrolled in the study are required to provide a signed informed consent document.

## Discussion

This is the first investigation of pharmacokinetics using a dose escalation trial with CBD in an athletic human population. There is limited data available on the dose escalation of CBD that examines the pharmacokinetics, physiological, and psychological profile. Furthermore, the majority of the literature reports on varying routes of administration, and pharmacokinetic research suggests that the half-life, absolute bioavailability, maximum serum concentration reached, and time to achieve maximum serum concentration vary depending on the dose [55].

The study product is given on a mg of CBD per kg body mass basis. Body mass can vary during the course of the study, but to allow for consistency and ability to supply individual dosing units to the participants, mg/kg doses will be based on the participant’s body mass determined during the Baseline Phase. The dose escalation proposed is based on the fact that, to date, there are no studies to suggest an appropriate dosing regimen for CBD products that is safe and tolerable for daily administration in contact sport athletes. Our proposed dosage escalation study, at six different dosages will be taken by all participants to allow us to assess the safety, efficacy and tolerability of the CBD at increasing concentrations. Literature review shows a wide range of CBD oral doses from 100 mg/day to 4500 mg/day have been administered safely [56–58]. Human studies using an acute single dosage of 6000 mg have not produced any serious or severe harmful effects [59]. The dose escalation and optimal dose has not been assessed to date. Consistent with the literature, a 2-week washout phase is used to ensure that no CBD remains in the body from any previous exposure prior to beginning the trial [60]. This will therefore be the first dose escalation CBD study that includes assessment of the optimal dose as assessed by pharmacokinetic activity.

Most studies also utilize arbitrary dosages (i.e., “shotgun approach”) or assess single dose parameters. Therefore, there is a need to understand the influences of varying doses of CBD on the physiological and pharmacological profiles, including the impact of dose escalation, when administered chronically (i.e., months). Ascending doses from 1500 to 6000 mg found that the maximum CBD concentration and half-life all vary depending on dose and the delivery of dose per day (such as twice daily as compared to a single-dose), with further evidence suggesting that the intake of a high-fat diet can increase the bioavailability of CBD by up to five-fold [59]. Therefore, given how different parameters associated with CBD dose and methods of administration all seem to influence its uptake, our first study will aim to address these concerns and provide guidelines for future research to base their designs.

Specific to sport-related concussion, the theoretical framework for the use of CBD as treatment to improved blood flow, cardiac parameters, and to provide neuroprotective benefits by enhancing the integrity of the blood brain barrier has been described [4]. Preliminary evidence was provided with a case series of 4 women with PPCS who showed improvements in cardiovascular parameters and concussion-related symptoms when administered CBD ranging from 50 to 400 mg daily, or a combined 20 mg CBD: 1 mg THC [39]. However, the evidence from a human clinical trial is still lacking. As such, our study will also assess how these physiological parameters are impacted in healthy male contact sport athletes (generally altered during concussion) and suggest an optimal dose for healthy participants to be implemented into a sports season in our future research.

While the study presents with fundamental strengths for assessing changes in physiological, neurophysiological and pharmacological activity, there are limitations associated with our methods. First, the study is not randomized, meaning there is potential for bias in our physiological results in association with higher CBD dosages. Furthermore, participants and researchers are not blinded to the condition, which can bias some of the outcomes. However, our primary outcome is the PK data, which will not be influenced by participant blinding. We are limited to assessing static and dynamic cerebral autoregulation. As such, we will not have an understanding of how cerebrovascular reactivity and neurovascular coupling are affected by CBD. While our participants are required to be at least 18 years of age, little is known about neurodevelopmental process from chronic CBD administration [61]. Regarding pharmacokinetic activity, we are hypothesizing that 2-weeks is sufficient to reach a steady state of CBD plasma concentrations, although research is currently lacking. Finally, given the large amount of time required per participant, we are unable to assess other aspects of cognitive and physical function, such as perceptual-cognitive and functional motor control assessments.

## Data Availability

No datasets were generated or analysed during the current study.
